# Cognitive Control of Intentions for Voluntary Actions in Individuals With a High Level of Autistic Traits

**DOI:** 10.1007/s10803-012-1509-9

**Published:** 2012-03-21

**Authors:** Edita Poljac, Ervin Poljac, Nick Yeung

**Affiliations:** 1 Department of Experimental Psychology, University of Oxford, South Parks Road, Oxford, OX1 3UD UK; 2 Laboratory of Experimental Psychology, University of Leuven, Leuven, Belgium; 3 Department of Psychology, International University of Sarajevo, Sarajevo, Bosnia and Herzegovina

**Keywords:** Autism, Autism-Spectrum Quotient, Cognitive control, Perseveration, Repetitive behavior, Voluntary task switching

## Abstract

Impairments in cognitive control generating deviant adaptive cognition have been proposed to account for the strong preference for repetitive behavior in autism. We examined if this preference reflects intentional deficits rather than problems in task execution in the broader autism phenotype using the Autism-Spectrum Quotient (AQ). Participants chose between two tasks differing in their relative strength by indicating first their voluntary task choice and then responding to the subsequently presented stimulus. We observed a stronger repetition bias for the harder task in high AQ participants, with no other differences between the two groups. These findings indicate that the interference between competing tasks significantly contributes to repetitive behavior in autism by modulating the formation of task intentions when choosing tasks voluntarily.

## Introduction

Human goal-directed behavior relies on neurocognitive control processes that allow for sustaining focus on tasks without being distracted, and for adapting to dynamically changing environmental conditions of daily life by shifting focus when necessary. This adaptive human cognition has often been studied in the lab using experiments in which participants rapidly switch between different tasks. From these studies we know that, although the ability to exert intentional control is not self evident as shown in different patient studies (e.g., Aron et al. 2004), the way it is expressed in behavior of typically developing individuals depends on a complex interaction between current intentions and past experiences (see e.g., Kiesel et al. 2010; Koch et al. 2010; Vandierendonck et al. 2010 for a review on behavioral findings; and Sakai 2008 for a review on findings from neuroimaging studies). Interestingly, while intentional control certainly provides the basis for cognitive flexibility, recent studies on task switching have reported empirical evidence for a consistent preference for repetitive voluntary behavior in healthy population. Specifically, when given an option to voluntarily choose which task to perform in each trial, while being encouraged to choose tasks at random and equally often, participants show a tendency to repeat tasks more often than to switch between them (e.g., Arrington and Logan 2004; Mayr and Bell 2006). Our study investigated how this repetition bias is expressed in healthy individuals with more autistic traits when given voluntary choice of tasks.

Repetitive behavior has been recognized as one of the key symptoms of autism spectrum disorders (ASD). Broadly speaking, this neurodevelopmental disorder is characterized by impaired social interaction and communication, and by restricted and repetitive behavior (APA 1994). Preference for repetitive behavior in individuals with autism has been reported both in everyday settings and clinical observations (e.g., extreme resistance to change of any kind) as well as in experimental settings (e.g., preservative responses on neuropsychological measures). Empirical evidence is mainly provided by various studies using a neuropsychological test—the Wisconsin Card Sorting Test (WCST)—in which the participant is required to sort different cards on the basis of three possible dimensions of the geometric figures depicted on the presented cards. The currently relevant sorting dimension is never explicitly given to the participant, and changes according to a fixed number of trials. The participant therefore needs to infer the sorting rule based on feedback provided by the experimenter and to decide whether to keep on applying the same rule or to change it. Performance on this test is measured in terms of errors, focusing in particular on perseverative errors that indicate trials in which participants maintain applying the previously relevant sorting rule, although the (error) feedback provided indicates that the rule has changed.

Many studies have shown that, relative to normally developing individuals and those with other neurodevelopmental disorders, individuals with autism exhibit highly perseverative responses in the WCST (see Hill 2004a, for a review). Based on these findings, it has been suggested that individuals with autism are cognitively inflexible, or more precisely, exhibit problems with switching between different thoughts or actions when required (Hill 2004b). Intriguingly, however, when tested in a more controlled experimental settings, this idea of deficits in cognitive flexibility as measured by deviant task switching performance is hardly supported by any empirical evidence (see Geurts et al. 2009 for a recent debate on this topic). For instance, Poljac et al. (2010) used task cues to unambiguously specify the required task in their study and reported that adolescents with autism switched between tasks in a similar way to their typically developing controls but significantly better than their clinical controls. This finding strongly implies that the deviations in behavior of individuals with autism as detected by the WCST cannot be accounted for in terms of an impaired ability to switch tasks, leaving an important issue to be addressed regarding the specific nature of the impaired mechanism reflected in behavior as a tendency to perseverate.

Considering that individuals with autism find it difficult to generate novel ideas and behaviors spontaneously (e.g., Boucher 1988; Craig and Baron-Cohen 1999; Turner 1999), it is not surprising then that putting demands on their intentional decision making in situations of undefined tasks—such as in the WCST—generates behavioral differences when compared with typically developing individuals. Following the same logic, reducing referential ambiguity in tasks (Preissler and Carey 2005) and directing of the intentions externally (Poljac et al. 2010) unsurprisingly facilitates their task performance such that it successfully eliminates behavioral differences. Accordingly, repeating tasks that are no longer appropriate might occur when the choice of the possible alternatives is not explicitly specified, leaving room for other factors to determine the task choice. For instance, recent studies on task switching using a voluntary procedure—in which participants are free to choose which task to perform on each trial—show that participants’ tendency to repeat tasks more often increases when the stimulus repeats (Arrington and Logan 2005; Mayr and Bell 2006; Yeung 2010). This finding suggests that bottom-up stimulus processing interacts with global intentional control as measured by task choice. The present study therefore aimed to investigate whether the observed tendency to exhibit repetitive behavior in autism could be explained in terms of bottom-up effects modulating the formation of global intentions when the tasks are unambiguously specified but the choice of which task to execute in a trial is voluntary.

To this aim, a double registration voluntary procedure was used in which participants make two responses on each trial: the first to register that they have made a choice of task by pressing a spacebar, the second to respond to the subsequently presented stimulus (see Millington et al. 2012, Experiment 2). Separating task choice from the actual task execution allows us to disentangle the participants’ global task intentions from their specific actions. Different studies have provided evidence that these two consider related yet dissociable processes (Arrington and Yates 2009; Butler et al. 2011; Mayr and Bell 2006; Yeung 2010). This distinction is important as it allows us to address the specific question of whether repetitive behavior in autism reflects intentional deficits rather than problems with implementing the task rule (action) once a task choice has been made (intention).

Of primary interest was to specifically investigate whether between-task interference would significantly contribute to the observed tendency to exhibit repetitive behavior in autism. A common way to elicit clear effects of between-task interference in task switching performance is by requiring participants to switch between tasks that differ in their relative strength. Under these conditions, the performance costs of task switching—as observed in slower response times (RTs) and higher error rates—shows an asymmetry: the switch costs seem to be greater for the easier task. This initially surprising, but now often replicated pattern of task switching performance in both instructed (e.g., Allport et al. 1994) and voluntary (e.g., Liefooghe et al. 2010; Yeung 2010) procedures clearly indicate a role of between-task interference in task execution. Interestingly, between-task interference has also been reported to affect participants’ choice of tasks. Specifically, the repetition bias seems to be stronger toward performing the more difficult task of a pair more often than the easier task (Liefooghe et al. 2010; Millington et al. 2012; Yeung 2010), implying a clear influence of between-task interference in the formation of task intentions. To induce between-task interference in our study we required the participants to voluntarily switch between a relatively easy location task and a relatively hard shape classification task.

Our participants were healthy individuals with either a low or a high number of autistic traits as measured by a self-report questionnaire that quantifies the extent of autistic traits in healthy population—the Autism-spectrum Quotient (AQ). The AQ has been used extensively to investigate the broader ASD phenotype with converging evidence that autism is not just a spectrum within the clinical population, but that autistic traits are continuously distributed through the general population (e.g., Baron-Cohen et al. 2001; Hoekstra et al. 2007). Many studies have shown that a higher position on the autism-like trait continuum of the AQ predicts cognitive processing similar to but often milder than that found in ASD (e.g., Bayliss et al. 2005; Fugard et al. 2011; Poljac et al. 2012; Ridley et al. 2011; Stewart et al. 2009; von dem Hagen et al. 2011). The AQ seems therefore to be sensitive to, and a useful tool for assessing, the broader ASD phenotype in non-clinical population (e.g., Bishop et al. 2004; Wheelwright et al. 2010).

In sum, this study was developed to test whether the tendency of individuals with autism to engage in repetitive behavior would also be detected in its broader phenotype assessed with the AQ, with the main focus on the question whether this repetitive behavior reflects intentional deficits rather than problems in task execution. We specifically tested the contribution of between-task interference to repetitive behavior in individuals with high level of autistic traits. We expected to find a stronger repetition bias in individuals with more autistic traits if the repetitive behavior in autism is mainly driven by between-task interference (captured in behavior as an asymmetry in registered measures) modulating the formation of task intentions (task choice). We furthermore expected to find no such difference in task performance (as measured in terms of switch costs) if the repetition behavior is not primarily driven by processes involved in eventual task execution (RTs and errors) even in a setting that requires flexible switching between tasks (cf. Poljac et al. 2010).

## Method

### Participants

Five hundred participants from the undergraduate psychology program at the University of Leuven took part in a pre-selection phase by completing the Autism-spectrum Quotient (AQ) questionnaire. The students participated voluntarily, for course credit and they all gave their written informed consent prior to the inclusion into the study. The protocol of the study was approved by the Ethics Committee of the Faculty of Psychology, University of Leuven, and was carried out in accordance with the ethical standards laid down in the 1964 Declaration of Helsinki.

### Autism-Spectrum Quotient (AQ)

The AQ is developed to estimate the presence and extent of autistic traits in healthy individuals, with scores ranging between 0 (low autistic traits) and 50 (high autistic traits). This questionnaire consists of 50 statements, for each of which four forced choices are offered to indicate whether participants ‘definitely agree’, ‘slightly agree’, ‘slightly disagree’, or ‘definitely disagree’ with each statement. The original administration of the test (Baron-Cohen et al. 2001) showed that 80 % of people with either Asperger syndrome (AS) or high-functioning autism (HFA) had a score between 32 and 50, whereas in a control group, only 2 % of people scored within that range. Based on this finding, the authors suggested the AQ as a valuable instrument for rapidly quantifying where any given individual is situated on the continuum from autism to normality. In this study, a Dutch version (translation by Ponnet et al. 2001) of the AQ (Baron-Cohen et al. 2001) was employed to quantify the amount of autistic traits in our participants. This AQ scale is highly comparable to the Dutch AQ scale validated by Hoekstra et al. (2008), and has already been successfully used by for instance Wouters and Spek (2011) who showed that this version had very high internal consistency in their typically developing participants (standarized Crohnbach’s alpha = 0.92).

### Stimuli and Tasks

Participants were presented with a shape (triangle, square, or circle) in one of three adjacent squares in a stimulus grid on each trial. At a viewing distance of approximately 60 cm, the stimulus grid was 2.6° high and 7.4° wide, and the presented shape approximately filled one square within the grid. The grid remained on the screen throughout the experimental block. Participants were required to voluntarily choose to respond either to the location or to the shape of each presented stimulus. Responding to the location involved deciding whether the stimulus appeared in left, center, or right location of the grid using a spatially compatible keypress. Responding to the shape included categorizing the shape identity with an arbitrarily mapped keypress. Stimulus shape and location varied randomly from trial to trial.

While making the task choice voluntarily, the participants were also encouraged to choose the two tasks at random and equally often. They were instructed to make their choice prior to stimulus presentation, and were reminded to do so by a cue consisting of the words ‘LOCATION/SHAPE’ appearing one above the other with large question marks on either side. Specifically, participants indicated that they had made a task choice (regardless of what that choice was) by pressing the spacebar. They then responded to the imperative stimulus using the hand appropriate for the chosen task. Half of the participants responded with their left hand for the shape task and their right hand for the location task. For the other half of the participants this mapping was reversed. Response keys were left/circle mapped to the leftmost finger of the responding hand, center/square mapped to the middle finger, and right/triangle to the rightmost finger.

### Procedure

Participants first completed the AQ questionnaire, and their individual AQ scores were used as a selection criterion for inclusion into the main experiment of voluntary task choice. The cutoffs were derived from the 5 % highest (AQ score > 24) and 5 % lowest scores (AQ score < 8) and only those who scored above or below these cut-offs were included in the main task (cf. Stewart et al. 2009). Accordingly, 25 students were assigned to each group, of whom 39 agreed to take part in the voluntary task-choice experiment. Specifically, 21 participants (14 female) with a score well above the average AQ (all scores above 24, mean score 28.5 ± 4.2) and 18 participants (13 female) who scored significantly below the average (all scores below 8, mean score 6.5 ± 0.8). Data of two participants (one from each group) were excluded from further analyses. One made 33 % errors on average, and the other failed to follow the given instructions.

The selected participants started the main experiment with three practice blocks of 50 trials, practicing first each task separately and then switching between the two tasks. In each trial, the participants chose which task to perform according to instructions taken from prior voluntary choice studies (Arrington and Logan 2004, 2005; Yeung 2010). Specifically, they were instructed to perform each task on about half the trials, and to try to perform the tasks in a random order, “as if flipping a coin that said ‘shape’ on one side and ‘location’ on the other”. They were furthermore explicitly instructed to make their choice before actually pressing the space bar.

Following practice, participants completed 6 blocks with voluntary choice procedure of 60 trials each. A trial started with the cue that appeared above the stimulus grid and remained there until the participant pressed the space bar indicating that they had decided which task to perform next. The imperative stimulus then appeared 300 ms later and remained on the screen until the response was given, followed by an interval of 500 ms showing the stimulus grid only. Both the choice of task and the actual response were not limited in time. At the end of each block, participants were given feedback showing their average response time (RT) and error rate as well as their task choices and the number of task switches and repetitions they made.

### Data Analysis

We analyzed the data focusing on two measures of task execution (RTs and error rates) and, critically, on two measures of task choice (participants’ actual choices and the speed with which they indicated these choices). All the measures were analyzed separately using repeated measures analyses of variance (ANOVAs), with task (location/shape) and transition type (switch/repeat) as within-subject factors, and AQ group (low/high) as between-subject factor. For the purpose of these analyses, each trial was first categorized according to task and transition type. Specifically, the task on a given trial was specified by the hand that the participant used to respond, and the transition type was determined according to the relation between the task performed on the current and the previous trial. A trial was coded as an error if the participant responded with the wrong finger of the used hand. Finally, analyses excluded the first trial of each block, and, for RT analyses, error trials and trials following errors.

## Results

To establish that the tasks differed in their relative difficulty as intended, and that patterns of switch costs would replicate the asymmetry previously reported in studies using the voluntary switching procedure, we first present data analysis of overall task execution. To furthermore establish that our participants used the cue period to make deliberative task choices, we then present analysis of choice speed before presenting the critical analyses of data of interest—participants’ task choices.

### Task Execution

Participants were on average both faster, *F* (1, 35) = 38.38; *p* < 0.01, and more accurate, *F* (1, 35) = 19.03; *p* < 0.01, when responding to the location of the stimulus (618 ms and 2.8 % errors) than when responding to its shape (752 ms and 5.8 % errors). This finding indicated the Location task as the relatively easier of the two, confirming the expected differences in task difficulty. The established task difficulty effect was similar in both AQ groups, *F* < 1 (see Table 1).

**Table 1:** Mean response time (ms), error rate (%), and choice time (ms) for low and high AQ participants as a function of task and transition type

	Low AQ participants (n = 21)			High AQ participants (n = 18)		
	Response time	Error rate	Choice time	Response time	Error rate	Choice time
Location						
Switch	800	4.6	418	652	3.1	455
Repeat	532	1.7	356	488	1.9	320
Switch cost	268	2.9	62	164	1.2	135
Shape						
Switch	844	5.6	455	764	7.1	512
Repeat	744	5.0	315	656	5.6	343
Switch cost	100	0.6	140	108	1.5	169

None of the effects significantly differed between the two groups

**Fig. 1:**
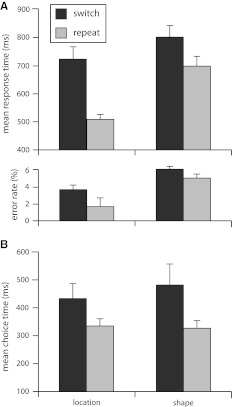
Task execution data and choice speed. *Panel A* depicts mean response times and error rates for the location and the shape task as a function of transition type (switch or repeat). *Panel B* depicts the mean time participants took before pressing the space bar as an indication that they have chosen the task to perform next. *Error bars* indicate SE of the mean

Furthermore, task execution on switch trials (766 ms and 5.1 % errors) was both slower, *F* (1, 35) = 33.95; *p* < 0.01, and more error-prone, *F* (1, 35) = 5.76; *p* = 0.02, than task execution on repeat trials (605 ms and 3.5 % errors), indicating clear switch costs in both RTs and errors, neither of which differed between the two AQ groups, *F*s < 1. Of interest here was to establish whether differences in task difficulty modulated task switching performance. The existence of this switch cost asymmetry was uncertain considering that the participants had unlimited time to make and indicate their task choices in our voluntary procedure. As shown in Fig. 1a, we indeed observed the asymmetry in the RT data, with a significant interaction between task and transition type, *F* (1,35) = 9.04; *p* < 0.01, indicating greater costs when switching to the easier location task than when switching to the harder shape task. No interaction was observed in the error data, *F* < 1. Importantly, the switch cost asymmetry was similar in both AQ groups, with the interaction between task, transition type, and AQ group not being significant either in RTs or in errors, *F*s (1,35) < 2.25; *p*s > 0.14 (see Table 1).

### Choice Speed

By analyzing the choice speed data, we aimed to assess whether the participants actively used the cue period to prepare themselves and to make deliberative task choices before hitting the space bar. Figure 1b shows how average choice speed was distributed across experimental conditions. Our data suggest that the participants indeed actively used the cue period, taking significantly more time to prepare for a task switch (460 ms) than a task repetition (333 ms), *F* (1,35) = 4.28; *p* < 0.05. As Fig. 1b illustrates, we furthermore observed a marginal interaction between task and transition type, *F* (1,35) = 3.74; *p* < 0.06, with the participants taking the most time to prepare the switch toward the shape task. Both the main effect of transition type and its interaction with task showed similar patterns in both AQ groups, *F*s < 1 (see Table 1).

### Task Choice

**Fig. 2:**
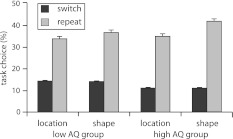
Percentage of task choice for the location and the shape task as a function of transition type (switch or repeat) in the low and high AQ participants. *Error bars* indicate SE of the mean

Analyzing the task choice data was of main interest in the present study, as it permitted testing the idea that the individuals with more autistic traits would show a stronger repetition bias, since repetitive behavior is considered to be one of the core characteristics of autism. We were therefore interested to see if the asymmetry in repetition bias, as previously observed in voluntary procedures using tasks that differ in their relative difficulty (e.g., Millington et al. 2012; Yeung 2010), would be stronger in our participants with high AQ scores. Consistent with this idea, we first replicated the general repetition bias, with participants choosing to repeat tasks more often (74 % of all trials) than choosing to switch tasks (26 % of all trials), *F* (1,35) = 53.31; *p* < 0.01. Furthermore, we replicated the asymmetry in repetition bias, with participants choosing to repeat the difficult shape task more often (40 % of all trials) than the easier location task (35 % of all trials), *F* (1,35) = 31.39, *p* < 0.01. Critically, however, a significant interaction between task, transition type, and AQ group was observed, *F* (1,35) = 4.70, *p* < 0.05. This finding indicates that although the asymmetry in repetition bias was present in both groups, with *F* (1,16) = 9.91, *p* < 0.01 and *F*(1,19) = 23.84, *p* < 0.01, for low and high AQ group respectively, the repetition bias towards the harder shape task was more strongly apparent for the participants having more autistic traits, as depicted in Fig. 2.

To further investigate our observation of a stronger asymmetry in repetition bias for high AQ participants, we analyzed an additional measure of repetitive behavior—the length of runs of trials participants make when given the voluntary choice of two tasks. Our replication of repetition bias implied already that our participants exhibited biases away from randomness although instructed to produce equal numbers of trials of each task and equal numbers of switch and repeat trials. Of particular interest here was to test whether the significantly stronger preference for repeating the harder shape task in high AQ participants as observed in percentages of task choices would also be observed as a tendency to produce longer runs for this task. The measure of run length is possibly the only way for a task preference to be expressed in voluntary procedures using two tasks, since the overall numbers of switches and runs when there are only two tasks must necessarily be roughly equivalent (Yeung 2010).

For the purpose of this analysis, we first categorized each trial by its position in the task run. We then calculated the logarithm of the average run of the two tasks for each participant. The logarithmic transformation was applied to correct for the differences in variance due to the skewed distribution of the run length. The analysis confirmed the general asymmetry in repetition bias also in this measure, *F* (1,35) = 29.77, *p* < 0.01, with participants making longer runs for the harder shape task (7.2 trials) than the easier location task (6.3 trials). Crucially, however, a significant interaction between task and AQ group, *F* (1,35) = 4.27, *p* < 0.05, confirmed that this tendency to produce longer runs in the harder task was significantly stronger in the participants with high AQ. Specifically, although the participants with low AQ also demonstrated the asymmetry in run length, *F* (1,16) = 11.23, *p* < 0.01, with the difference between the shape and the location task being 0.7 trials, the participants with high AQ showed a significantly stronger tendency to produce longer runs of trials in the harder task, with the difference being 1.1 trials in this group, *F* (1,19) = 21.33, *p* < 0.01.

Repetitive behavior seems to be a complex construct reflected in behavior of individuals with autism across multiple distinct facets (e.g., Lam et al. 2008; Langen et al. 2011). Interestingly, a subset of items (10 out of 50) included in the AQ is related to restrictive and repetitive behaviors—*Attention switching domain*, as described in Hoekstra et al. (2008). We therefore used these scores to test their possible contribution to repetitive behaviors as measured in our voluntary procedure. We first counted the scores that each of our participants had on the items belonging to the Attention switching domain, measuring repetitive behavior. We then calculated the relative contribution of these items to the total AQ score as a difference between this actual score and their expected contribution to the total AQ score (i.e., expected was 20 % of the total score). The relative contribution of repetitive behavior as measured by the AQ was significantly higher than 0 in both high AQ, with average relative contribution of 1.13, *t*(19) = 3.12, *p* = 0.006, and in low AQ participants, with average relative contribution of 0.92, *t*(16) = 3.07, *p* = 0.007. Moreover, the average contribution of repetitive behavior did not significantly differ between the groups, *t*(35) = −0.44, *p* = 0.66. Interestingly, when taken as a covariate, the items measuring repetitive behavior did not modulate the general tendency to choose the harder task more often or to make longer runs for this task, *F*s < 1. Analyzing each of the AQ groups separately confirmed the general observation: the repetitive behavior ratio did not significantly influence task choice or run length in high AQ participants, *F*s < 1, nor in low AQ participants, *F* (1,15) = 1.01, *p* = 0.33 and *F* < 1, respectively.

Collectively, our task choice data demonstrated a significantly stronger asymmetry in repetition bias for the participants with more autistic traits in both of its measures—percentages of task choice and run length. The two measures are highly correlated in general (r = 0.91; *p* < 0.001), and when tested in both of the tasks separately (r = 0.90; *p* < 0.001; and r = 0.88; *p* < 0.001; for the location and the shape task, respectively). Task choice and run length were not significantly modulated by the items within the AQ scale related to restrictive and repetitive behaviors. The final analysis we performed on the task choice data aimed to further investigate the processes contributing to the observed asymmetry in repetition bias. Previous studies have already shown that repeating tasks is in general facilitated by stimulus repetition (Mayr and Bell 2006; Yeung 2010), suggesting that bottom-up stimulus processing affects people’s intentions and contributes to the repetition bias observed in voluntary procedures. Although our participants were instructed to make their choice during the cue period and to indicate to have made the choice by pressing the space bar, which then initiated the stimulus presentation, it is still possible that their final task choices were affected by the subsequent stimulus processing such that they disregarded their initial intentions. Just recently, Millington et al. (2012, Experiment 2) used a similar space-bar voluntary procedure and observed a tendency in their participants to occasionally ignore the initial intention and repeat the shape task more often when the shape of the stimulus repeated. We therefore aimed to test if our observation of the stronger asymmetry in repetition bias in people with more autistic traits was possibly generated or in some way affected by bottom-up stimulus processing. Table 2 shows how the proportion of task repetitions were distributed over different types of stimulus repetitions (shape, location, both, or neither repeated) for both low and high AQ participants. We only observed a marginal interaction between AQ group and repetition of stimulus shape, *F* (1,35) = 3.41, *p* = 0.07, with the proportion of task repetitions not being significantly different when stimulus shape repeated (0.80) from when it changed (0.79) in participants with high AQ, *F* (1,19) = 2.41, *p* = 0.14, whereas the participants with low AQ repeated tasks more when the stimulus shape repeated (0.76) than when it changed (0.71), *F* (1,16) = 7.41, *p* < 0.05. This finding indicates that the stronger asymmetry in repetition bias towards the harder shape task observed in high AQ participants did not simply arise from stimulus repetition. Furthermore, repetition of stimulus location only generally increased the proportion of task repetitions, *F* (1,35) = 7.02, *p* < 0.05, from 0.75 to 0.78, showing no interaction with AQ group, *F* < 1.

**Table 2:** Proportion of task repetitions as a function of whether the stimulus location and stimulus shape repeated from the previous trial

	Location changes		Location repeats	
	Shape changes	Shape repeats	Shape changes	Shape repeats
Low AQ participants				
Just performed location	0.70	0.75	0.71	0.76
Just performed shape	0.71	0.73	0.72	0.80
High AQ participants				
Just performed location	0.74	0.77	0.80	0.81
Just performed shape	0.78	0.79	0.82	0.83

## Discussion

The primary aim of this study was to investigate the mechanism behind the tendency to exhibit repetitive behavior reported in autism. To this end, we compared behavioral patterns in voluntary task choice in healthy individuals varying in their level of autistic traits as measured by the AQ. The findings demonstrate a significantly stronger tendency to specifically repeat the harder task more often for the participants with high level of autistic traits. Consistent with other studies showing that the AQ predicts various cognitive abilities similar to that found in ASD (e.g., Fugard et al. 2011), these results confirm that a comparable bias toward repetitive behavior—one of the main symptoms of ASD—can also be detected when measuring voluntary task choices in the broader autism phenotype as assessed by the AQ.

Critically, these findings suggest that the repetition bias in autism arises from processes involved in the formation of general task intentions rather than from those involved in task execution: While the patterns of task choice—both the proportion of task choices as well as the run length—showed a stronger bias toward repeating the harder task in participants with more autistic traits, no differences in behavior during actual task execution were found between high and low AQ participants. Consistent with previously reported patterns of task switching performance in autism (e.g., Poljac et al. 2010; Schmitz et al. 2006; Shafritz et al. 2008; Stahl and Pry 2002; Whitehouse et al. 2006), the two AQ groups showed no differences in switch-specific performance. Specifically, both groups replicated earlier results from studies using voluntary procedure in a similar way by showing a reliable switch cost (e.g., Arrington and Logan 2004) as well as a reliable asymmetry in switch cost (e.g., Yeung 2010). Since the behavioral patterns of the two AQ groups were overall similar, showing no differences in any of the measures other than participants’ task choice, it seems highly unlikely that the stronger asymmetry in repetition bias observed in high AQ group developed from some kind of a general impairment in their task performance. In fact, high AQ participants were in general numerically faster in task execution (640 vs. 730 ms for high and low AQ, respectively), showed numerically smaller switch costs (136 vs. 183 ms) as well as a numerically smaller switch cost asymmetry (56 vs. 168 ms). These patterns strongly suggest that the specific differences observed in repetition bias are not simply generated by some general discrepancy in task performance between the two groups.

The observed asymmetry in participants’ switch costs and task choices replicated previous findings in studies using voluntary task switching procedures (e.g., Millington et al. 2012; Yeung 2010). These findings clearly indicate the existence of interference in our study. As expected, this interference between the two competing tasks affected the processes involved in task execution (switch cost asymmetry) and in task intentions (task repetition asymmetry). Importantly, however, the observation of a significantly stronger asymmetry in task repetitions in participants with more autistic traits suggests that the strong preference for repetitive behavior in autism is most probably generated by interference between the competing tasks modulating the formation of task intentions. Just recently, Poljac and Yeung (2012) provided neural evidence for the involvement of between-task interference in intentional task preparation when tasks are chosen voluntarily, confirming these and other behavioral data (e.g., Arrington and Logan 2005; Mayr and Bell 2006; Millington et al. 2012; Orr and Weissman 2011; Yeung 2010). Poljac and Yeung’s data suggest that between-task interference modulates global task intentions rather than either motor-related preparation or the actual task execution. It thus seems that between-task interference in our study affects the formation of intentions for voluntary actions in people with high level of autistic traits more than it does in those with less autistic traits.

This remarkable tendency to repeat the harder task of a pair has been suggested to reflect differences in control biases being stronger in the harder task (cf. Gilbert and Shallice 2002; Yeung and Monsell 2003). According to this idea, the persisting biases toward the harder task increase the difficulty of switching away from this task to the easier task, leading participants to exhibit a surprising preference for performing the harder task (Yeung 2010). Following this line of reasoning, our findings suggest that the difference in control biases between the two tasks was larger in participants with more autistic traits. The stronger biases required for the harder task were possibly additionally enhanced in our high AQ group, creating a significantly stronger attractor state that is less likely to decay over time, so that, once the choice for this harder task has been made, these participants were more inclined to continue performing this overall more effortful task. In other words, the difference in repetitive behavior observed in our study seems to be generated by a dynamic interaction of differences in control biases that individuals varying across the dimension of autistic traits have for tasks, influencing the formation of task intentions when the task choice is voluntary.

This account would also predict the observed perseverative behavior in the WCST (e.g., Liss et al. 2001; Ozonoff and Jensen 1999), in which the interplay of control biases for tasks become especially important since the tasks are not defined to the participants in advance. Accordingly, when the appropriate rule—defined by the experimental procedure—has been selected and reinforced by feedback, this task develops a strong attractor state making it hard to abandon it if the rule implicitly changes. Since in the WCST task alternatives are not specified, abandoning this highly preferred task then becomes particularly difficult in individuals with ASD who seem to be more sensitive to the influence that differences in control biases have on the formation of intentions for voluntary actions. Directing intentions by providing cues, on the other hand, aids task execution in individuals with ASD, reflected in behavior as an overall similar pattern between them and their healthy controls (e.g., Poljac et al. 2010). The influence of control biases in these instructed designs would be expected to become apparent as stronger switch cost asymmetries in individuals with ASD. An interesting analogy comes from studies examining memory functioning in autism reporting that, while cued-recall paradigms tend to yield no deviant performance (e.g., Bowler et al. 1997), free-recall paradigms generally lead to diminished performance in this population (e.g., Gaigg et al. 2008; Smith et al. 2007), emphasizing again the importance of intentional directing in autism. Our findings are also in line with the idea of an open versus closed system account of autism proposed by Lawson et al. (2003, 2004). According to this view, individuals with autism are biased to approach the world as a closed system, that is, as a system in which degrees of freedom are minimized. According to Lawson and colleagues, this bias is why they experience difficulties with tasks that are more open (i.e., include more variability). In this type of task, top-down intentions are more crucial, such is the case in the voluntary procedure used in our study.

It is worthy of note here that, similar to the observation reported by Millington et al. (2012) in their Experiment 2, all participants tended to be particularly slow in making the choice of switching to the more difficult shape task, even though they exhibited an overall bias toward choosing this task more often. Participants also took more time to choose task switches compared to task repetitions (see also Arrington and Logan 2005; Orr and Weissman 2011). In both conditions they took more time to choose—task switching in general and switching to the harder task in particular—suggesting that our choice time findings might reflect participants’ preference for minimizing effort (e.g., Arrington 2008; Arrington and Yates 2009; Lien and Ruthruff 2008). This preference for less effort is possibly also reflected in the observed effects between task choice and stimulus processing. Similar to what other studies using voluntary procedure have already reported (e.g., Arrington an Logan 2005; Mayr and Bell 2006; Yeung 2010), our participants were in general inclined to repeat the task at hand more often if the stimulus repeated, suggesting that external bottom-up stimulation occasionally determined the eventual task choice. Importantly, however, this bottom-up influence of stimulus processing on task choice seems not to be the driving mechanism behind the stronger asymmetry in repetition bias detected in the participants with more autistic traits: The only difference between the two groups observed when analyzing the effects of stimulus repetition on task choice revealed that, whereas task choices of high AQ participants were not affected by repetitions of stimulus shape, the participants with low level of autistic traits were more prone to repeat the task at hand on these occasions. This finding evidently implies that the stronger bias toward repeating the shape task in high AQ participants is not generated by differences in effects of stimulus repetitions between the two groups.

Three additional issues regarding our findings need further elaboration. First, the high number of female participants in our study reflected the gender distribution in the population we tested—undergraduate psychology students. Although additional analyses demonstrated that none of our findings were significantly affected by gender (*F*s < 1), the predominantly female population makes it harder to generalize our findings to the autism population for two reasons: the prevalence of autism is higher in males, and male and female individuals with autism seem to differ in behavior in various aspects (e.g., Lai et al. 2011). Whether our findings are generalizable to female individuals with autism only would need to be addressed in future research.

Second, instructions used in voluntary task switching paradigms typically stress the requirement to choose tasks randomly and equally often. The instruction regarding balanced task choices could require participants to keep track of which tasks they have performed in past, putting high demands on their working memory. Some studies have reported that individuals with autism are challenged on working memory tasks (e.g., Geurts et al. 2004; Zinke et al. 2010). It could therefore be possible that the observed differences in our study were predominantly due to the challenge that our high AQ participants experienced with keeping track of task history. Interestingly, however, Butler et al. (2011) recently demonstrated that working memory capacity is more strongly related to task performance than to task choice in voluntary task switching. If our AQ groups differed in the way that they were challenged by high working memory demands present in the task we used, then according to Butler et al., this should have been reflected in the performance measures (RTs and error rates) of the two groups. We observed no such differences. Although we cannot exclude the possibility that working memory contributed to the observed differences between high and low AQ participants, Butler et al.’s study and our observations in task performance together would suggest that our findings would need a broader explanation than one including working memory only.

Third, it is possible that some other important factors, such as intelligence, modulated the patterns of behavior in the population that we tested. One could imagine that the numerically better performance—observed in the high AQ participants—was generated by a higher IQ in these individuals. Since we had no a priori reasons to assume that our student population would differ in their IQ, we did not assess any measures of their intelligence. We therefore cannot exclude the possibility that intelligence significantly contributed to the observed differences between our groups. To our knowledge, there are no studies so far reporting a relation between IQ and performance on voluntary task switching paradigms. Interestingly, however, the few studies that have tested a possible contribution of IQ to observed differences between high and low AQ individuals seem not to find any dependence of the AQ effects on IQ in children (Auyeung et al. 2009) or in adults (students) for verbal (Stewart and Ota 2008) or full IQ measures (Grinter et al. 2009). The contribution of IQ to behavioral patterns observed in voluntary task switching paradigms would need to be specified in future research.

In conclusion, the present study indicates that between-task interference significantly contributes to the tendency of individuals with autism to engage in repetitive behavior by modulating the formation of task intentions when tasks are chosen voluntarily. Our results reveal that the way in which between-task interference modulates global task intentions—as measured by behavioral patterns in task choice—depends on the quantification of where an individual lies along the dimension of autistic traits. On the contrary, the way that task execution—as measured by behavioral patterns in RTs and errors—is affected by this interference between the competing tasks seems not to be related to the amount of autistic traits in healthy individuals.
